# GALNT2 promotes invasiveness of colorectal cancer cells partly through AXL

**DOI:** 10.1002/1878-0261.13347

**Published:** 2022-12-05

**Authors:** Ying‐Yu Liao, Ya‐Ting Chuang, Hsuan‐Yu Lin, Neng‐Yu Lin, Tzu‐Wen Hsu, Szu‐Chia Hsieh, Syue‐Ting Chen, Ji‐Shiang Hung, Hung‐Jen Yang, Jin‐Tung Liang, Min‐Chuan Huang, John Huang

**Affiliations:** ^1^ Graduate Institute of Anatomy and Cell Biology, College of Medicine National Taiwan University Taipei Taiwan; ^2^ Department of Medical Research National Taiwan University Hospital Taipei Taiwan; ^3^ Department of Anatomy, College of Medicine Chang Gung University Taoyuan Taiwan; ^4^ Department of Surgery National Taiwan University Hospital Taipei Taiwan; ^5^ Min‐Sheng General Hospital Taoyuan Taiwan

**Keywords:** AXL, GALNT, glycosylation, invasion

## Abstract

GalNAc‐type O‐glycosylation and its initiating GalNAc transferases (GALNTs) play crucial roles in a wide range of cellular behaviors. Among 20 GALNT members, *GALNT2* is consistently associated with poor survival of patients with colorectal cancer in public databases. However, its clinicopathological significance in colorectal cancer remains unclear. In this study, immunohistochemistry showed that GALNT2 was overexpressed in colorectal tumors compared with the adjacent nontumor tissues. GALNT2 overexpression was associated with poor survival of colorectal cancer patients. Forced expression of GALNT2 promoted migration and invasion as well as peritoneal metastasis of colorectal cancer cells. In contrast, GALNT2 knockdown with siRNAs or knockout with CRISPR/Cas9 system suppressed these malignant properties. Interestingly, we found that GALNT2 modified O‐glycans on AXL and determined AXL levels via the proteasome‐dependent pathway. In addition, the GALNT2‐promoted invasiveness was significantly reversed by AXL siRNAs. These findings suggest that GALNT2 promotes colorectal cancer invasion at least partly through AXL.

## Graphical abstract

TAM (TYRO3, AXL, and MERTK) receptors regulate a wide range of pathophysiological functions. Here, we show that TAM receptors are decorated with O‐glycans. GALNT2 can modify O‐glycans on AXL to increase AXL protein levels and in turn promote colon cancer cell invasiveness. This study highlights the role of GalNAc‐type O‐glycosylation in TAM receptor biology.

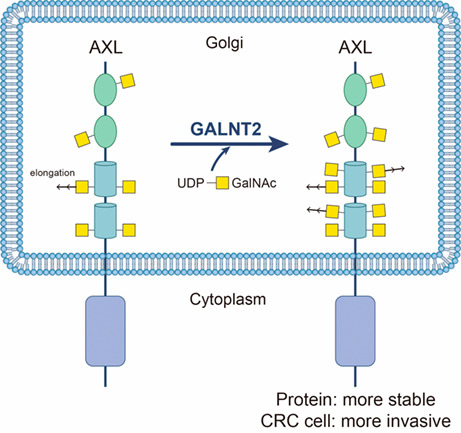

**Abbreviations**
ATCC: American type cell culture collection
CQ: chloroquine
EGFR: epidermal growth factor receptor
EMT: epithelial–mesenchymal transition
FN: fibronectin
GalNAc: *N*‐acetylgalactosamine
GALNT: GalNAc transferase
HCC: hepatocellular carcinoma
ICD: intracellular domain
Ig: immunoglobulin
IHC: immunohistochemistry
KO: knockout
MT1‐MMP: membrane type‐1 matrix metalloproteinase
RTK: receptor tyrosine kinase
RT‐PCR: reverse transcription‐polymerase chain reaction
sgRNA: single‐guide RNA
STR: short tandem repeat
TCGA: The Cancer Genome Atlas
VVA: *Vicia Villosa* lectin

## 1 Introduction

Colorectal cancer is the second leading cause of cancer death [1]. It develops over several years, starting as benign adenomatous polyps becoming advanced adenoma with high‐grade dysplasia, and then progresses to invasive cancer [1]. The 5‐year survival rate of patients with metastatic colorectal cancer is decreased by eight times compared to patients with local disease [2]. Colorectal cancer patients with peritoneal metastasis have worse overall survival than those with other isolated sites of metastasis such as liver or lung [3]. There is an urgent need to find new diagnostic markers and identify therapeutic targets to manage metastatic colorectal cancer.

The TAM family of receptor tyrosine kinases (RTKs) consists of TYRO3, AXL, and MERTK, which are activated by their ligands GAS6 and Protein S [4]. All three TAM members share similar structures – the kinases contain two immunoglobulin‐like (Ig‐like) domains and two fibronectin (FN) domains in the N‐terminal region, while the tyrosine kinase domain is toward the C terminus [4]. They are expressed by many cell types and are often co‐expressed by various cells with functional redundancy [5]. The TAM receptors are emerging as important regulators of cell proliferation and survival processes in cells as well as key components of immunity, inflammation, and cancer. Abnormal overexpression of TAM receptors and/or GAS6 has been documented in a wide range of malignancies [6]. Recently, the TAM receptors represent a group of novel anticancer targets due to their promotion of tumor cell survival, proliferation, invasion, and chemoresistance, as well as suppression of the immune status of the tumor microenvironment [7].

Altered glycosylation affects many cellular properties, including cell proliferation, apoptosis, differentiation, adhesion, migration, invasion, and immune responses [8]. Tumor‐associated carbohydrate antigens have been shown to predict poor survival in several cancers and attracted much attention to developing diagnostic agents and vaccines for cancer therapy [9, 10]. Two major forms of protein glycosylation in mammalian cells are N‐linked and O‐linked. The most abundant type of O‐glycosylation is GalNAc‐type O‐glycosylation, initiated by the transfer of *N*‐acetylgalactosamine (GalNAc) to the hydroxyl group of a serine (S) or threonine (T) residues to form Tn antigen (GalNAcα‐S/T) [11]. This reaction is catalyzed by a large family of polypeptide GalNAc transferases (GALNTs), which consists of 20 members in humans, namely, GALNT1 to 20 [10, 12]. GALNT enzymes are differentially expressed in various tissues in a spatiotemporal‐dependent manner [12].

Many reports have demonstrated the importance of GalNAc‐type O‐glycans and *GALNT* genes in a wide range of biological functions and cancers [10]. Changes in O‐glycans on β1‐integrin modulate integrin signaling and alter cellular properties [13]. O‐glycosylation regulates autolysis of cellular membrane type‐1 matrix metalloproteinase (MT1‐MMP), which is a key enzyme in cancer invasion and metastasis [14]. MUC1 is an oncoprotein in many human cancers and can be O‐glycosylated by GALNT1, 2, and 3 [15]. GALNT6 can suppress the progression of colorectal cancer [16]. In addition, GALNT6 has been reported as a diagnostic marker of breast cancer [17] and may regulate tumorigenesis of breast cancer by modulating O‐glycosylation of fibronectin [18]. GALNT14 regulates death‐receptor O‐glycosylation and controls tumor‐cell sensitivity to the proapoptotic ligand Apo2L/TRAIL [19]. Our team found that GALNT2 is downregulated in hepatocellular carcinoma (HCC) and its re‐expression suppresses the malignant character of HCC by modifying the O‐glycosylation and activity of EGF receptor [20]. Moreover, GALNT2 suppresses malignant phenotypes through IGF‐1 receptor and predicts favorable prognosis in neuroblastoma [21]. By contrast, GALNT2 enhances migration and invasion of oral squamous cell carcinoma by regulating EGFR glycosylation and activity [22]. Although we have found that GALNT2 plays either tumor‐suppressive or tumor‐promoting role in several cancer types, its pathophysiologic functions in colorectal cancer remain unclear.

## 2 Materials and methods

### 2.1 Patient samples

Fifty‐eight patients who had undergone surgery in the period between 2005 and 2010 at the National Taiwan University Hospital were selected for this study. Patients' information is shown in Table S1. Written consent was obtained from the patients; the hospital's Institutional Review Board approved this study (IRB No: 202009095RINA). Subsequently, paraffin‐embedded tissue blocks of colorectal adenocarcinomas and their surrounding nontumor tissues were collected.

### 2.2 Immunohistochemistry

The paraffin‐embedded tissue section was incubated with an anti‐GALNT2 antibody (1 : 100, Sigma, MO, USA) at 4 °C for 16 h. Super Sensitive™ Link‐Label IHC Detection System (BioGenex, Fremont, CA, USA) was used and signals were visualized through a 3,3‐diaminobenzidine (DAB) liquid substrate system (Sigma, St. Louis, MO, USA). The tissues were counterstained with hematoxylin and mounted with UltraKitt (J.T. Baker, Deventer, Holland). Negative controls were performed by replacing the primary antibody with a control IgG at the same concentration. The intensity of immunohistochemical staining was scored by two independent researchers who were blinded to the clinical data of patients. The scores were categorized into four groups as follows: 0 (negative), 1 (weakly positive), 2 (moderately positive), and 3 (strongly positive).

### 2.3 Cell lines and cell culture

SW620 and HCT116 cell lines were authenticated using short tandem repeat (STR) profiling analysis. Caco‐2, COLO205, HCT15, HCT116, HT29, LoVo, SW480, SW620, and MDA‐MB‐231 cells were maintained in a complete DMEM medium containing 10% fetal bovine serum (FBS; Life Technologies, Burlington, Canada) and 1% penicillin/streptomycin (P/S; Gibco, Invitrogen™, Thermo Fisher Scientific, Waltham, MA, USA), and cultured at 37 °C in air with 5% CO_2_. All cell lines were obtained from American Type Culture Collection (Manassas, VA, USA).

### 2.4 cDNA synthesis and real‐time RT‐PCR

Total RNA from cells was isolated using TRIzol reagent (Invitrogen) according to the manufacturer's instructions, as described previously [23]. Briefly, 2 μg of total RNA was used for cDNA synthesis in a 20 μL reverse transcription (RT) reaction using High‐Capacity cDNA Reverse Transcription Kits (AB, USC). The real‐time PCR reactions were performed in 20 μL volume containing 1 μL cDNA, 10 μL SensiFAST SYBER Lo‐ROX Mix (BIOLINE, Meridian Life Science, Inc., Memphis, TN, USA), and primer pairs using QuantStudio 3 Real‐Time PCR system (Thermo). Primers for AXL were 5′‐AACCAGGACGACTCCATCC‐3′ and 5′‐AGCTCTGACCTCGTGCAGAT‐3′ and primers for GAPDH were 5′‐GACAAGCTTCCCGTTCTCAG‐3′ and 5′‐ACAGTCAGCCGCATCTTCTT‐3′.

### 2.5 Transfection and plasmid construction

For transient knockdown, two independent GALNT2‐specific siRNAs (Invitrogen) and nontargeting siRNA (Invitrogen; Dharmacon, Lafayette, CO, USA) were used to transfect colon cancer cells through Lipofectamine RNAiMAX (Invitrogen) with a final concentration of 10 nm for 2 days. The siRNAs against GALNT2 were si‐GALNT2‐1: 5′‐CAGCAGGGAACUAACUGCCUCGACA‐3′ and si‐GALNT2‐2: 5′‐UGUCGAGGCAGUUAGUUCCCUGCUG‐3′. The siRNAs against AXL were si‐AXL‐1: 5′‐GAGCUGCGGGAAGAUUUGGAGAACA‐3′ and si‐AXL‐2: 5′‐CCAGGAACUGCAUGCUGAAUGAGAA‐3′. The nontargeting siRNAs (si‐Control) were 5′‐CAACCUCAGCCAUGUCGACUGGUUU‐3′. For stable knockdown, shGALNT2‐1/pLentiLox 3.7 (Target sequence: GTAGAAATGATCGACGAGA) and its empty control pLentiLox 3.7 plasmid were used. For stable overexpression of GALNT2 in colon cancer cells, we used a lentiviral infection system. In brief, lentiviruses were harvested from the culture supernatant of HEK293FT cells transfected with 20 μg GALNT2/TRIPdU3IRES‐GFP or its empty TRIPdU3IRES‐GFP plasmid, 15 μg psPAX2, and 6 μg VSVG. HCT116 and SW620 cells were infected with recombinant lentivirus. After 48 h, GFP‐expressing cells were isolated by fluorescence sorting using a FACSArialIII cell sorter (Becton Dickinson, Mountain View, CA, USA). Overexpression of GALNT2 in the pooled clones was confirmed by western blot analysis. In addition, the positivity rate of the stable clones with GALNT2 overexpression was analyzed by immunofluorescence microscopy using an anti‐GALNT2 primary antibody (Sigma) and a goat anti‐rabbit IgG secondary antibody conjugated with Alexa Fluor 594 (Thermo Fisher Scientific).

### 2.6 Western blot analysis

Proteins were separated on an 8% SDS/PAGE gel and transferred onto a PVDF membrane. After blocking with 5% bovine serum albumin (BSA; Bio‐Rad, Hercules, CA, USA) for 1 h at room temperature, membranes were incubated with primary antibodies at 4 °C overnight. Antibody against GAPDH was purchased from Santa Cruz Biotechnology (Santa Cruz, CA, USA). Antibodies against AXL, p‐AXL, TYRO3, MERTK, EGFR, p‐EGFR, MET, p‐MET, p‐AKT, p‐ERK, and ERK were purchased from Cell Signaling Technology (Danvers, MA, USA). Antibody against AKT was purchased from GeneTex Inc. (Irvine, CA, USA). The membranes were then incubated with horseradish peroxidase‐conjugated secondary antibodies, and proteins were detected using ECL reagents (GE Healthcare Life Sciences, Piscataway, NJ, USA).

### 2.7 MTT assay

Cell viability was analyzed using MTT assay as described previously [23]. Briefly, colon cancer cells (1.5 × 10^3^) in 100 μL of complete DMEM were seeded in 96‐well plates for 16 h, and then 10 μL of 5 mg·mL^−1^ 3‐(4,5‐dimethyl‐2‐thiazolyl)‐2,5‐diphenyl‐2H‐tetrazolium bromide solution (MTT; Sigma) was added to each well for the indicated times and incubated at 37 °C for 3 h, and the MTT formazan crystals were dissolved with 100 μL 10% SDS containing 0.01 N HCl. The resultant optical density was measured spectrophotometrically at the dual wavelengths of 550 and 630 nm.

### 2.8 Transwell migration and Matrigel invasion assays

Cell migration and invasion were evaluated using transwell (Corning, Steuben, NY, USA) and Matrigel‐coated (BD Biosciences, San Jose, CA, USA) transwell chamber, respectively. Each transwell chamber contained a membrane of pore size 8 μm. SW620 (1 × 10^6^) and HCT116 cells (1 × 10^5^) in 0.25 mL serum‐free DMEM were seeded into the upper part of transwell or Matrigel‐coated transwell chamber. The lower chamber was filled with 0.5 mL DMEM containing 10% FBS as a source of chemoattractants. After incubation for 24 or 48 h, the cells were fixed and stained with 0.5% (w/v) crystal violet (Sigma) in 20% (v/v) methanol. The migrated and invaded cells from three random fields were counted under a microscope.

### 2.9 Lectin pull‐down assay

To analyze changes in O‐glycans, 300 μg of total cell lysates was incubated with *Vicia Villosa* lectin (VVA) agarose beads (Vector Laboratories, Burlingame, CA, USA) at 4 °C overnight. After washing with PBS twice, the pulled‐down proteins were subjected to western blot analysis.

### 2.10 Knockout of GALNT2 using CRISPR/Cas9 system

To knock out *GALNT2*, the CRSPR/Cas9 system was used. The single‐guide RNA (sgRNA; target sequence: CGTAAGGGTCCTGCCCGGAG) and surrogate plasmid of *GALNT2* gene were designed by National RNAi Core Facility at Academia Sinica in Taiwan. The pAll‐Cas9.Ppuro plasmid co‐expressed Cas9 and sgRNA of *GALNT2*. A pSurrogate reporter plasmid contained an sgRNA target sequence between an in‐frame EGFP cassette and an out‐of‐frame mCherry cassette. Briefly, HCT116 cells at a density of 1 × 10^6^ cells per well in a six‐well plate were co‐transfected with 2.5 μg of pAll‐Cas9.Ppuro and 2.5 μg of pSurrogate reporter plasmids using Lipofectamine 3000 kit (Invitrogen). At 48 h after transfection, viable mCherry‐positive cells were sorted using a 4‐laser FACSAriaIII sorter (BD Biosciences) and then cultured in 96‐well plates. Single colonies of HCT116 cells with successful *GALNT2* knockout were confirmed by DNA sequencing and western blotting.

### 2.11 Confocal microscopy

Cells were cultured in chamber slides (SPL Life Sciences, Gyeonggi‐do, Korea), fixed with 4% PFA, and permeabilized with 0.25% Triton X100. After blocking in PBS containing 2% bovine serum albumin (Bio‐Rad), cells were incubated with a primary antibody against AXL (Cell Signaling Technology). An isotype‐matched antibody was used as a control. Alexa Fluor antibody (Thermo Fisher Scientific) was used as the secondary antibody. Cell nuclei were visualized with DAPI (Santa Cruz Biotechnology). Images were captured using a confocal microscope (Carl Zeiss LSM880 with Airyscan, Jena, Germany).

### 2.12 Protein degradation analysis

To analyze proteasome‐dependent degradation, cells were treated with 1 μm proteasome inhibitor MG132 (Sigma) and its solvent control DMSO at 37 °C for 0, 2, 4, and 6 h. For analysis of lysosome‐dependent degradation, 10 or 20 μm chloroquine diphosphate salt (Sigma) and its solvent control PBS were used to treat cells at 37 °C overnight. AXL and the loading control GAPDH were detected using western blot analysis.

### 2.13 *In vivo* xenograft animal model

For *in vivo* peritoneal metastasis analysis, 1 × 10^7^ of HCT116 cells or 2 × 10^7^ of SW620 cells were intraperitoneally injected into 5‐week‐old male nonobese diabetic/severe combined immunodeficiency (NOD/SCID) mice (National Laboratory Animal Center, Taiwan). Animal experiments were reviewed and approved by the Institutional Animal Care and Use Committee (IACUC) of College of Medicine, National Taiwan University (IACUC Approval No: 20180428). All mice used in this study were maintained under specific pathogen‐free conditions and housed in pathogen‐free facilities in a 12 h light/dark cycle with *ad libitum* access to food and water. Animal studies were conducted in strict accordance with the recommendations in the Guide for the Care and Use of Laboratory Animals.

### 2.14 Statistical analysis

Statistical analyses were performed using ibm spss statistics 20 (Chicago, IL, USA) and GraphPad Prism 6 (GraphPad Software, Inc., San Diego, CA, USA). Student's *t*‐test was used to compare differences between the two experimental groups. Overall survival was compared through log‐rank comparisons for time‐to‐event data using Kaplan–Meier methods. Student's *t*‐test was used to analyze *in vitro* and *in vivo* experiments. Two‐sided *P* < 0.05 was considered statistically significant.

## 3 Results

### 3.1 GALNT2 is overexpressed in colorectal tumors and is associated with poor survival

**Fig. 1:**
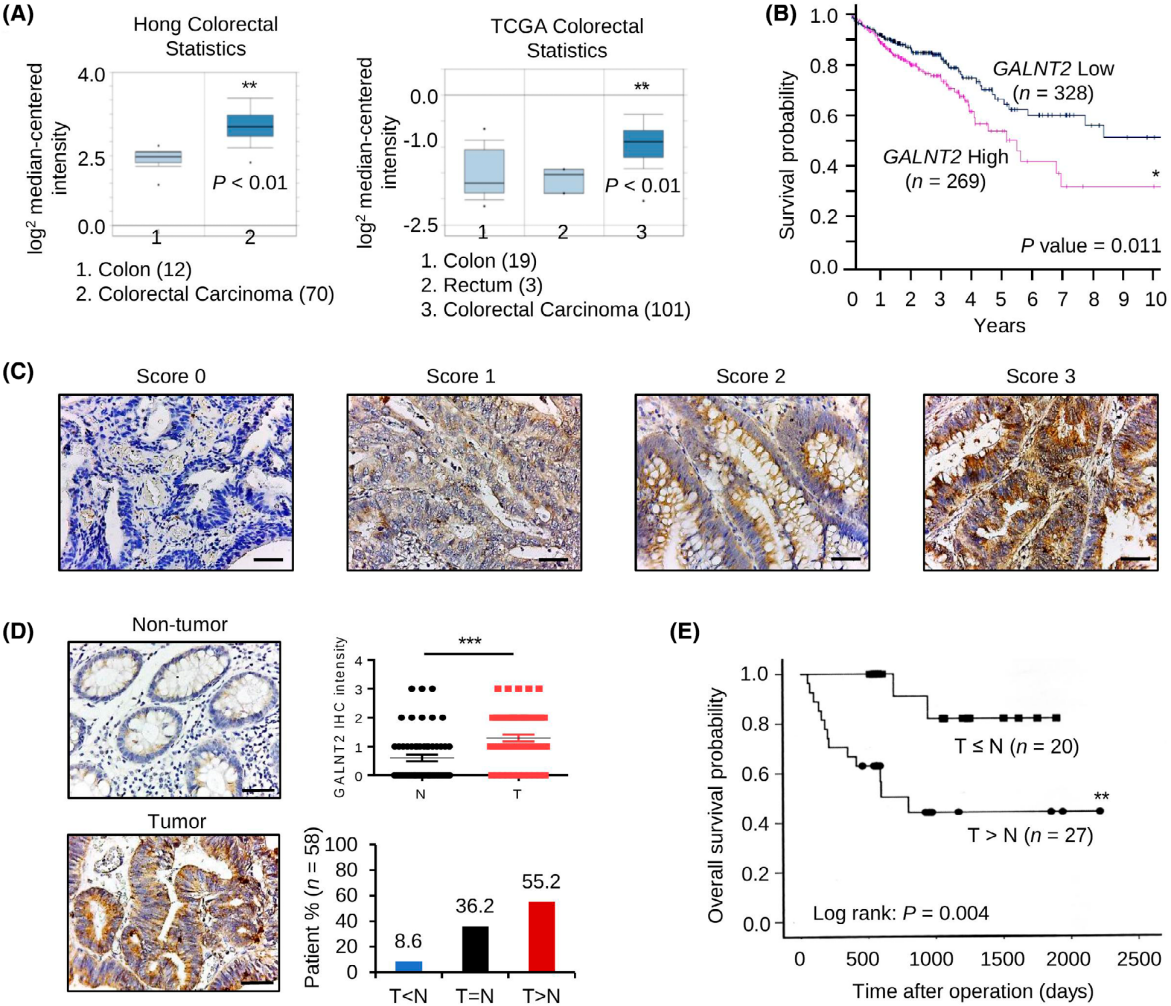
GALNT2 is overexpressed in colorectal tumors and is associated with poor survival. (A) *GALNT2* mRNA levels in normal and cancerous colorectal tissues in the Oncomine database. Hong colorectal statistics: 82 samples; TCGA colorectal statistics: 123 samples. Data are presented as mean ± SEM. (B) Kaplan–Meier survival curve of colorectal cancer patients based on *GALNT2* mRNA levels generated using the Human Protein Atlas database. The cut‐off value of low and high *GALNT2* expression is 14.64. *GALNT2* low and high expression groups contain 328 and 269 patient samples, respectively. (C) Scores of GALNT2 expression were analyzed using immunohistochemistry. GALNT2 in colorectal tumors and adjacent nontumor tissues was immuno‐stained with an anti‐GALNT2 antibody using immunohistochemistry. Representative images showing the staining intensity from score 0 to 3. *n* = 58. Scale bars, 50 μm. (D) GALNT2 was frequently overexpressed in colorectal tumors (T) compared with their surrounding nontumor tissues (N) analyzed by immunohistochemistry (IHC). Scale bars, 50 μm. The statistical results of GALNT2 IHC in pared colorectal tumor tissues (*n* = 58) are shown. Data are presented as mean ± SEM. ***P < 0.001 using paired Student's *t*‐test. (E) Kaplan–Meier analysis shows that GALNT2 overexpression correlated with poor survival of patients with colorectal cancer. *n* = 47. *P < 0.05, **P < 0.01, ***P < 0.001.

Data retrieved from the Human Protein Atlas and Kaplan–Meier Plotter indicated that, among 20 GALNT enzymes, only *GALNT2* mRNA levels are significantly associated with poor survival of patients with colorectal cancer in both databases (Table 1). By contrast, *GALNT3*, *GALNT5*, and *GALNT6* are associated with favorable outcomes. We, therefore, focused on the investigation of the clinical significance of GALNT2 in colorectal cancer. Data from the Hong and TCGA Colorectal Statistics indicated that *GALNT2* is overexpressed in colorectal tumors compared with the normal colorectal tissues (Fig. 1A). Kaplan–Meier survival curves using TCGA dataset in the Human Protein Atlas showed that high *GALNT2* expression is associated with poor survival of patients with colorectal cancer (Fig. 1B). Next, we analyzed clinical samples of colorectal cancer from the National Taiwan University Hospital using immunohistochemistry. The staining intensity of GALNT2 was scored from 0 to 3 (Fig. 1C). We found that GALNT2 protein was overexpressed in colorectal tumors compared with their adjacent nontumor tissues (Fig. 1D). Kaplan–Meier analysis showed that overexpression of GALNT2 is associated with poorer overall survival of patients with colorectal cancer (Fig. 1E). These results suggest that GALNT2 is overexpressed in colorectal tumors and its overexpression is associated with poor survival.

**Table 1:** Kaplan–Meier 5‐year overall survival analysis of *GALNT* gene family in colorectal cancer. HR is shown only when the *P* value of survival is less than 0.05 in both databases. CI, confidence interval; HR, hazard ratio; NA, not available.

Gene name	The Human Protein Atlas (*n* = 597)	Kaplan–Meier plotter (*n* = 165)	
	*P* value	*P* value	HR (CI 95%)
*GALNT1*	0.044*	0.0587	
*GALNT2*	0.011*	0.0042*	3.12 (1.37–7.10)
*GALNT3*	0.001*	0.0080*	0.36 (0.17–0.79)
*GALNT4*	0.088	0.0137*	
*GALNT5*	0.021*	0.0090*	0.36 (0.16–0.80)
*GALNT6*	0.023*	0.0073*	0.27 (0.10–0.74)
*GALNT7*	0.000*	0.1739	
*GALNT8*	0.011*	NA	
*GALNT9*	0.150	0.0179*	
*GALNT10*	0.099	0.1898	
*GALNT11*	0.050	0.2514	
*GALNT12*	0.230	0.1478	
*GALNT13*	0.025*	0.2085	
*GALNT14*	0.021*	0.1799	
*GALNT15*	0.064	0.2185	
*GALNT16*	0.400	0.0766	
*GALNT17*	0.055	0.1046	
*GALNT18*	0.037*	0.1480	
*GALNT19*	NA	NA	
*GALNT20*	NA	NA	

* *P* value < 0.05 is considered statistically significant.

### 3.2 GALNT2 overexpression promotes migration and invasion of colon cancer cells

**Fig. 2:**
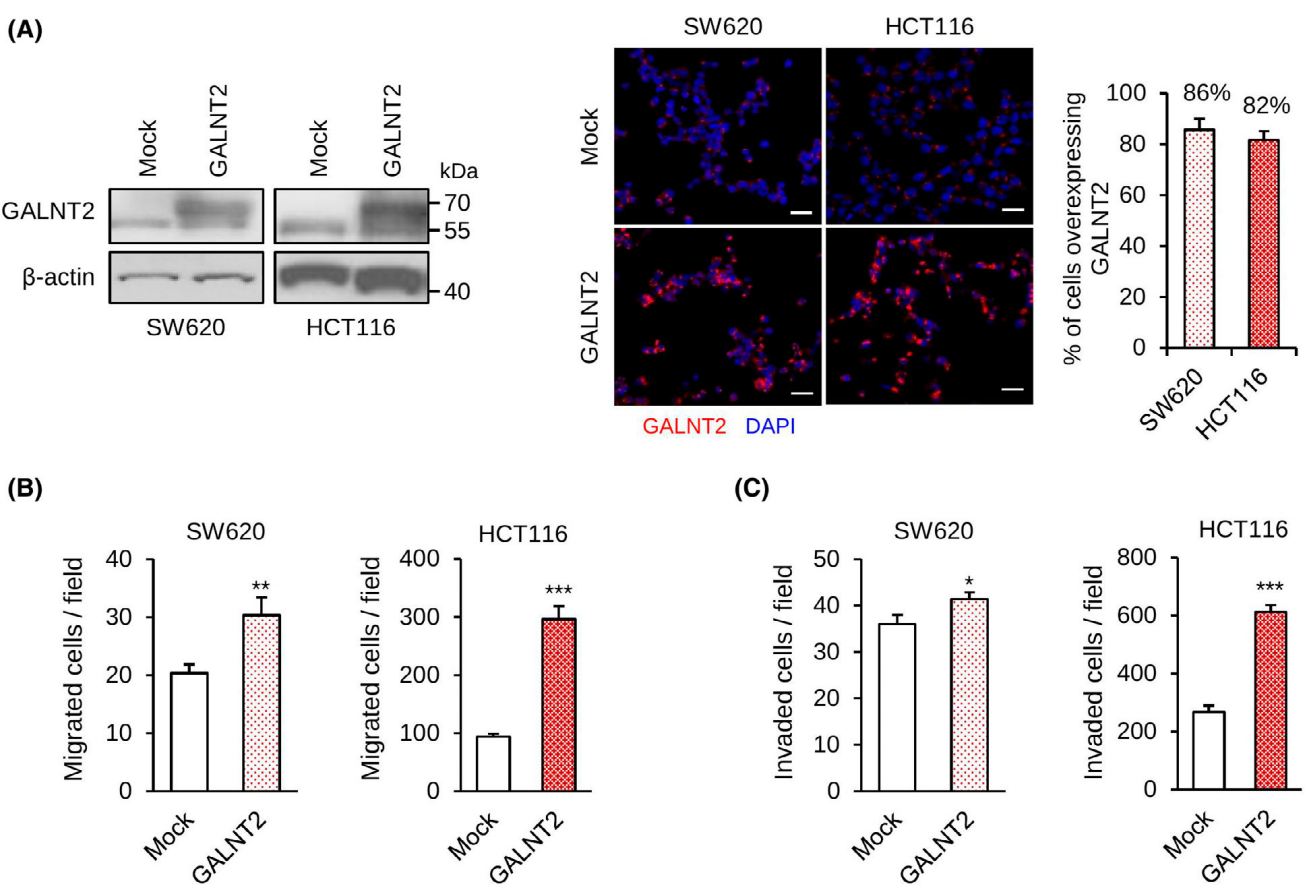
GALNT2 overexpression promotes migration and invasion of colon cancer cells. (A) GALNT2 overexpression was confirmed using western blotting and immunofluorescence microscopy. Western blots showing GALNT2 expression in mock or GALNT2 overexpressing SW620 and HCT116 cells. β‐actin is an internal control. Representative images of immunofluorescence microscopy showing the high percentage of cells with GALNT2 overexpression in SW620 and HCT116 cells stably transfected with GALNT2 plasmid. GALNT2 was stained in red. Nuclei were stained with DAPI (blue). Five random fields of each pooled cell were calculated. Scale bars, 20 μm. Data are presented as mean ± SD. *n* = 3. (B) Transwell migration assays. (C) Matrigel invasion assays. Data are presented as mean ± SD. *n* = 3. *P < 0.05 and **P < 0.01 using Student's *t*‐test.

Because metastasis is a critical determinant of cancer deaths, we therefore focused on the effect of GALNT2 on colon cancer cell migration and invasion, which are important *in vitro* phenotypes of metastasis. First, western blot analysis confirmed that GALNT2 was stably overexpressed in the pooled clones of SW620 and HCT116 cells (Fig. 2A). In addition, immunofluorescence microscopy indicated that GALNT2 was overexpressed in 86% and 82% of SW620 and HCT116 stable transfectants, respectively. Transwell migration assays indicated that GALNT2 overexpression enhanced the migration of SW620 and HCT116 cells (Fig. 2B). Moreover, Matrigel invasion assays indicated that GALNT2 overexpression enhanced the invasion of SW620 and HCT116 cells (Fig. 2C). MTT viability assays showed that GALNT2 overexpression increased cell viability of HCT116 but not SW620 cells (Fig. S1). These results suggest that GALNT2 promotes migration and invasion of colon cancer cells.

### 3.3 GALNT2 knockdown inhibits invasiveness of colorectal cancer cells

**Fig. 3:**
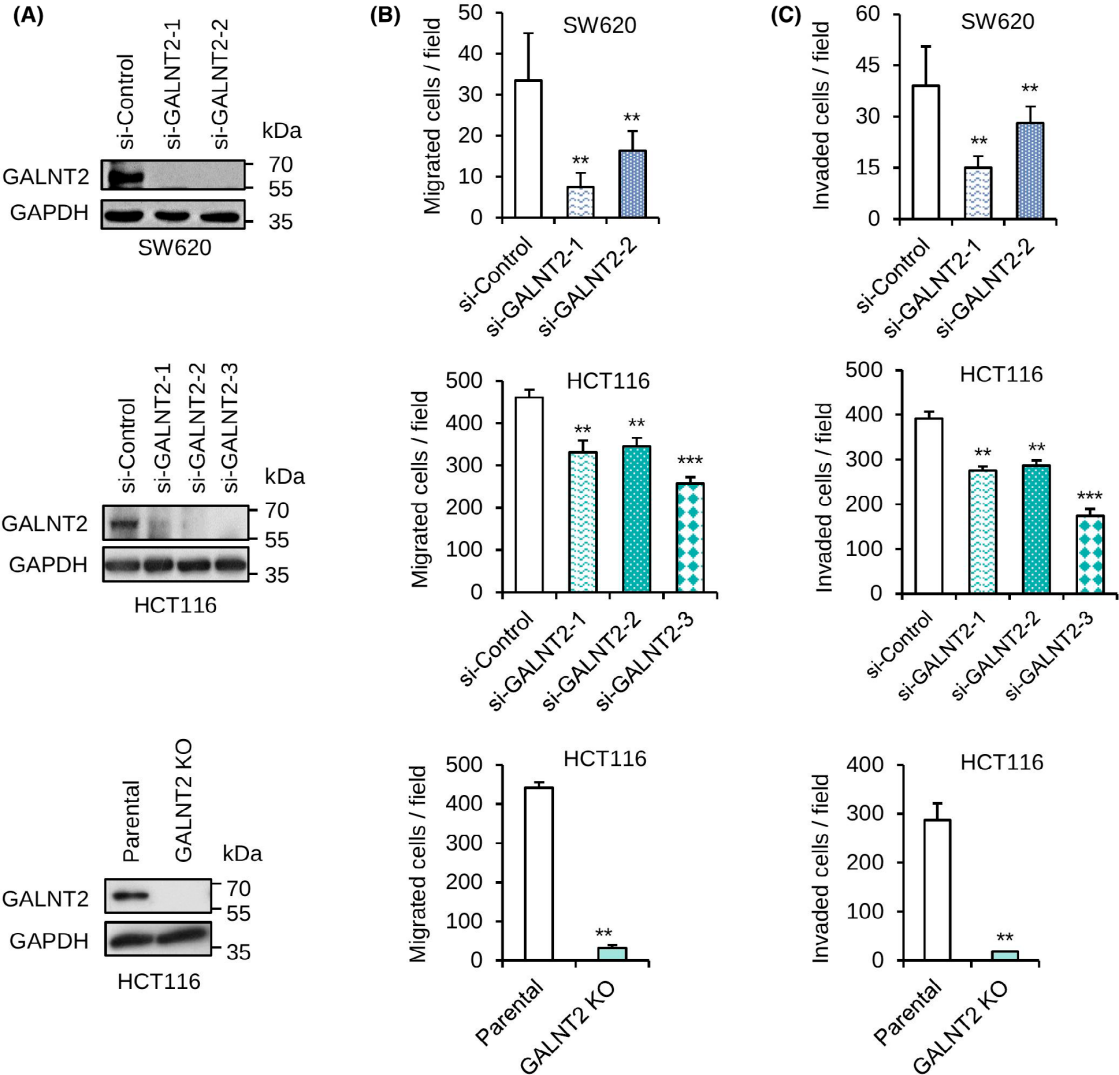
GALNT2 knockdown or knockout inhibits invasiveness of colon cancer cells. (A) Western blots showing GALNT2 knockdown in SW620 and HCT116 cells as well as GALNT2 knockout (KO) in HCT116 cells. *n* = 3. GALNT2 was knocked down with different siRNAs. GALNT2 was knocked out using the CRISPR/Cas9 system. (B) Cell migration was analyzed using transwell migration assay. (C) Cell invasion was analyzed by Matrigel invasion assay. Data are presented as mean ± SD. *n* = 3. **P < 0.01 and ***P < 0.001 using student's *t*‐test.

To further confirm the effect of GALNT2 on migration and invasion of colon cancer cells, GALNT2 was knocked down with different siRNAs or knocked out with the CRISPR/Cas9 system in SW620 and HCT116 cells (Fig. 3A). The results from transwell migration assay showed that GALNT2 knockdown or knockout suppressed migration of SW620 and HCT116 cells (Fig. 3B). Matrigel invasion assays showed that GALNT2 knockdown or knockout suppressed invasion of SW620 and HCT116 cells (Fig. 3C). Moreover, we also demonstrated that GALNT2 knockdown suppressed migration and invasion of SW480 cells (Fig. S2). These results suggest that silencing or loss of GALNT2 expression is sufficient to suppress migration and invasion of colon cancer cells, including SW620, HCT116, and SW480.

### 3.4 Effects of GALNT2 on peritoneal tumor metastasis in NOD/SCID mice

**Fig. 4:**
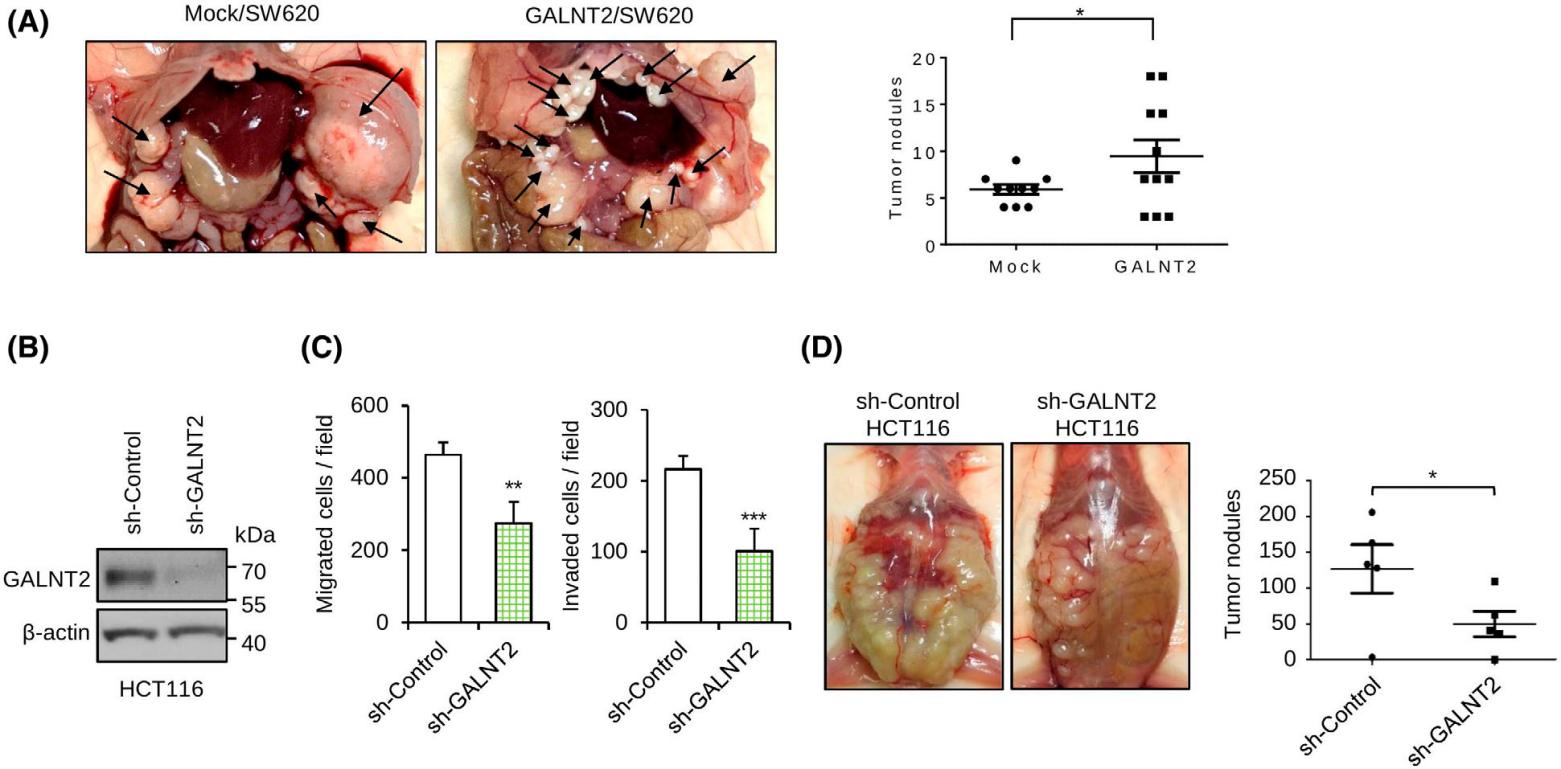
Effects of GALNT2 on invasive behaviors of colon cancer cells and peritoneal metastases in NOD/SCID mice. (A) Representative images of tumor formation in NOD/SCID mice intraperitoneally injected with control (mock) and GALNT2 overexpressing (GALNT2) SW620 cells. Mice were sacrificed on day 90. Arrows indicate tumors. Statistical results of tumor nodule numbers are presented as mean ± SD. *n* = 11 for each group obtained from two experiments. (B) Western blots showing stable knockdown of GALNT2 in HCT116 cells. Nontargeting shRNA (sh‐control) was used as control for GALNT2‐specific shRNA (sh‐GALNT2). *n* = 3. (C) Transwell migration and Matrigel invasion assays. *n* = 3. (D) Representative images of tumor formation in NOD/SCID mice intraperitoneally injected with HCT116 cells knocked down with sh‐control or sh‐GALNT2. Mice were sacrificed on day 30. Right panel, statistical results of tumor nodule numbers are presented as mean ± SD. *n* = 5 for each group. *P < 0.05, **P < 0.01, ***P < 0.001. Statistical data were analyzed and obtained through Student's *t*‐test.

To investigate the effect of GALNT2 overexpression on tumor peritoneal metastasis *in vivo*, SW620 cells with or without GALNT2 overexpression were intraperitoneally injected into NOD/SCID mice. The results showed that GALNT2 overexpression significantly (*P* = 0.037) increased the number of peritoneal tumor nodules (Fig. 4A). To know the effect of GALNT2 knockdown on peritoneal metastasis of colon cancer cells, GALNT2 was stably knocked down in HCT116 cells (Fig. 4B). Consistently, stable knockdown of GALNT2 suppressed migration and invasion of HCT116 cells *in vitro* (Fig. 4C). In the mouse model, GALNT2 knockdown significantly decreased the number of peritoneal tumor nodules (Fig. 4D). These results suggest that GALNT2 promotes peritoneal metastasis of colon cancer cells, whereas silencing of GALNT2 is sufficient to suppress the peritoneal metastasis *in vivo*.

### 3.5 GALNT2 knockout only weakly affects EGFR and MET signaling

Because we and others have ever found that GALNT2 could regulate EGFR and MET phosphorylation [22, 24, 25, 26] which have been demonstrated to modulate colorectal cancer metastasis, we tested if GALNT2 knockout could interfere with their signaling. Our data showed that GALNT2 knockout only weakly decreased EGF‐triggered phosphorylation of EGFR and HGF‐triggered phosphorylation of MET (Fig. S3). To further test whether EGFR and MET contribute to GALNT2‐mediated migration and invasion, mock or GALNT2 overexpressing HCT116 cells were treated with an EGFR‐specific inhibitor erlotinib or a MET‐specific inhibitor PHA665752. Western blot analysis confirmed that erlotinib and PHA665752 suppressed phosphorylation of EGFR and MET, respectively (Fig. S4A). However, transwell migration and Matrigel invasion assays showed that erlotinib or PHA665752 could not suppress migration and invasion of HCT116 cells (Fig. S4B,C). We even found that erlotinib and PHA665752 to a lesser extent could enhance migration and invasion of HCT116 cells. Taken together, these results suggest that EGFR and MET play a little, if any, role and are dispensable in GALNT2‐mediated migration and invasion in HCT116 cells. These findings imply that, in addition to EGFR and MET, GALNT2 may regulate colon cancer cell invasiveness through other molecules.

### 3.6 GALNT2 adds O‐glycans to AXL and regulates AXL protein levels

**Fig. 5:**
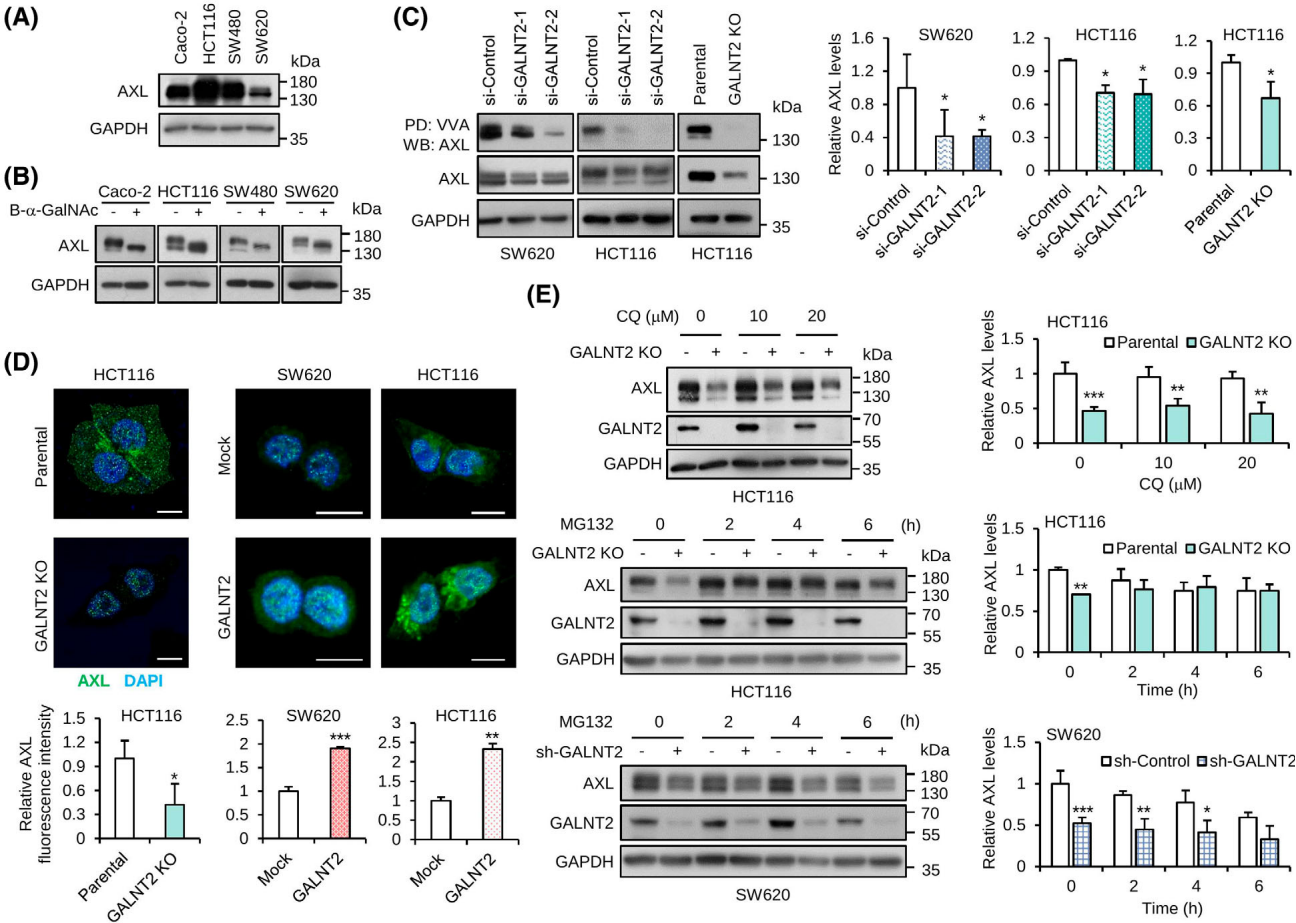
GALNT2 adds O‐glycans to AXL and regulates AXL protein levels. (A) Western blots showing the expression of AXL in colon cancer cell lines. GAPDH was a loading control. *n* = 3. (B) Western blots showing the effect of O‐glycan biosynthesis inhibitor on the molecular weight of AXL. Cells were treated with 2 mm benzyl‐α‐GalNAc (B‐α‐GalNAc) for 24 h. *n* = 2. (C) VVA pull‐down assay. AXL was pulled down with VVA‐agarose beads and then immunoblotted for AXL. GAPDH was a loading control. Western blotting signals were quantified using imagej software. Statistical data showing relative AXL pulled down by VVA‐agarose beads after normalization to input AXL. *n* = 3. (D) Confocal microscopy of AXL. Colon cancer cells as indicated were stained with an anti‐AXL antibody followed by FITC‐conjugated secondary antibody. Nuclei were stained with DAPI. Scale bar, 20 μm. The relative intensity of AXL signals was quantified using five cells from each image. (E) Western blot analysis of AXL in cells treated with lysosome inhibitor chloroquine (CQ) or proteasome inhibitor MG132. Parental and GALNT2 knockout (KO) HCT116 cells as well as mock and stable GALNT2 knockdown (sh‐GALNT2) SW620 cells were used. Relative AXL levels normalized to GAPDH were quantified using ImageJ software (National Institutes of Health, Bethesda, Maryland, USA.) and are shown. Data are presented as mean ± SD. *n* = 3 for each group. *P < 0.05, **P < 0.01, ***P < 0.001, by Student's *t*‐test.

Next, we analyzed the protein sequence of receptor tyrosine kinases (RTKs) and realized that the TAM family (TYRO3, AXL, and MERTK) of RTKs have fibronectin domains. Fibronectin is known to be heavily decorated GalNAc‐type O‐glycans [27]. Among the TAM receptors, AXL was consistently reported to play a crucial role in the invasive behavior of cancers, including colorectal cancer [27]. We, therefore, hypothesized that AXL carries O‐glycans and GALNT2 initiates the biosynthesis of the O‐linked glycans. First, western blot analysis showed that expression levels of AXL varied in Caco‐2, HCT116, SW480, and SW620 cells (Fig. 5A). To demonstrate whether AXL is decorated with GalNAc‐type O‐glycans, colon cancer cells were treated with benzyl‐α‐GalNAc to inhibit O‐glycan elongation. The results showed that benzyl‐α‐GalNAc decreased the molecular weight of AXL in Caco‐2, HCT116, SW480, and SW620 cells (Fig. 5B), suggesting that AXL indeed carries O‐glycans. Because all TAM receptors share similar structures, we analyzed if TYRO3 and MERTK are also decorated with O‐glycans. Our data showed that benzyl‐α‐GalNAc treatment decreased the molecular weight of TYRO3 and MERTK (Fig. S5), suggesting that O‐glycans are also present in TYRO3 and MERTK in colon cancer cells.

To demonstrate that GALNT2 can add O‐glycans on AXL, we performed *Vicia Villosa* Lectin (VVA) pull‐down assay. VVA is known to specifically recognize GalNAc on serine or threonine residues. Our results showed that GALNT2 knockdown in SW620 and HCT116 cells as well as GALNT2 knockout in HCT116 cells significantly decreased GalNAc on AXL (Fig. 5C). These results suggest that the GalNAc‐type O‐glycans on AXL can be added by GALNT2 in colon cancer cells.

In the western blots, we found that AXL protein levels were decreased by GALNT2 knockdown or knockout. To further confirm the effect of GALNT2 on AXL protein levels, confocal microscopy was performed. Consistently, the results showed that GALNT2 knockout significantly decreased AXL expression in HCT116 cells (Fig. 5D). By contrast, GALNT2 overexpression significantly increased AXL expression in both SW620 and HCT116 cells, although prominent changes were mainly observed in the cytoplasm. Real‐time RT‐PCR analysis indicated that GALNT2 knockout even slightly increased *AXL* mRNA expression (Fig. S6). In addition, GALNT2 knockdown or overexpression did not significantly affect *AXL* expression. These results suggest that the changes in the AXL protein levels by GALNT2 did not result from altered *AXL* mRNA levels. To know whether GALNT2 regulates AXL expression through lysosomal or proteasomal degradation pathway, cells were treated with chloroquine or MG132, respectively. The results of western blot analysis showed that MG132, but not chloroquine, blocked the significant effect of GALNT2 knockout or knockdown on AXL degradation in HCT116 and SW620 cells (Fig. 5E), suggesting that GALNT2‐mediated O‐glycosylation modulates AXL degradation primarily through the proteasomal degradation pathway. To know whether GALNT2 can regulate ligand‐mediated activation of AXL, GAS6 was used to treat mock and GALNT2 overexpressing SW620 cells. Although phosphorylation of AXL and AKT in MDA‐MB‐231 breast cancer cells was evidently induced by GAS6, the phosphorylation of AXL in SW620 did not show a significant elevation in our experimental conditions (Fig. S7). Collectively, our data demonstrated that GALNT2 can generate O‐glycans on AXL and regulate AXL protein levels in colon cancer cells.

### 3.7 AXL is involved in GALNT2‐promoted invasiveness in colon cancer cells

**Fig. 6:**
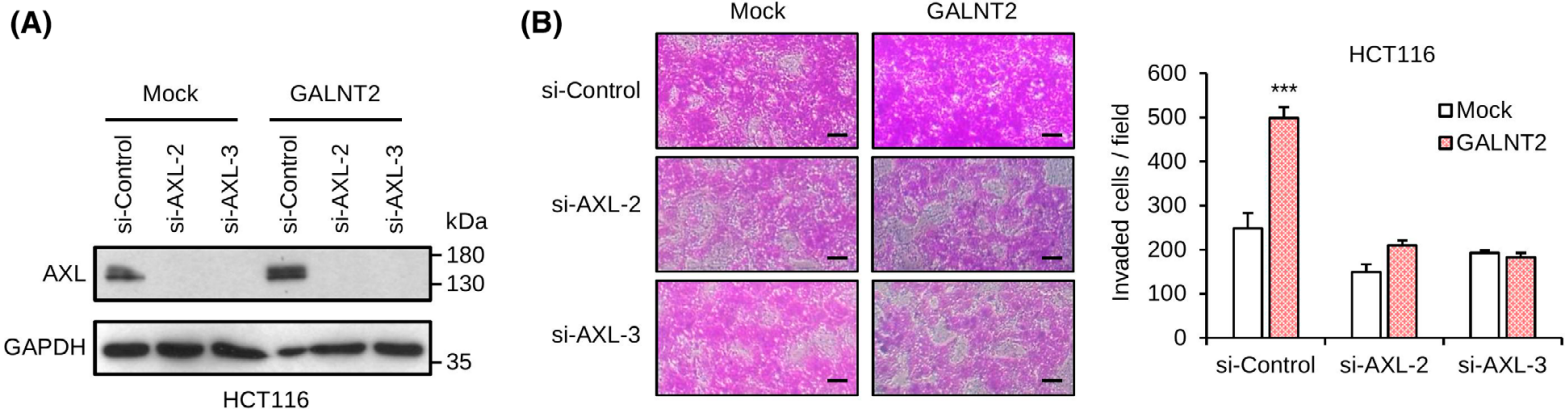
GALNT2‐promoted invasiveness is primarily through AXL in colon cancer cells. (A) Western blots showing siRNA‐mediated knockdown of AXL in GALNT2 stable overexpressing HCT116 cells. Two independent siRNAs against AXL (si‐AXL‐2 and si‐AXL‐3) were used. *n* = 3. (B) Effects of AXL siRNAs on GALNT2‐promoted invasion of HCT116 cells. Representative images of the Matrigel invasion assay are shown. Scale bar, 20 μm. Data are presented as mean ± SD. *n* = 3, ***P < 0.001, by Student's *t*‐test.

To evaluate if AXL is involved in GALNT2‐mediated invasiveness, Matrigel invasion assays were performed in mock and GALNT2 overexpressing SW620 and HCT116 cells treated with or without an AXL inhibitor or siRNAs. Cells were treated with TP‐0903, which can inhibit all TAM receptors, but is much more selective for AXL [21, 28]. The results showed that TP‐0903 was able to significantly reverse GALNT2‐mediated invasion in SW620 and HCT116 cells (Fig. S8). To have more specific inhibitory effect on AXL, cells were treated with two different siRNAs against *AXL* in mock or GALNT2 overexpressing HCT116 cells. Consistent with the results from TP‐0903 treatment, GALNT2‐mediated invasion was significantly reversed by AXL siRNAs (Fig. 6). Taken together, these results suggest that AXL is involved in GALNT2‐promoted invasiveness in colon cancer cells. Because TYRO3 and MERTK are also decorated with O‐glycans, we tested whether TYRO3 and MERTK can contribute to the GALNT2‐mediated effect. We found that TYRO3 or MERTK siRNA significantly reversed HCT116 cell invasion (Fig. S9). We next examined the effect of GALNT2 knockdown on TYRO3 and MERTK protein levels. The results showed that GALNT2 knockdown also decreased TYRO3 and MERTK protein levels (Fig. S10). These results suggest that TYRO3 and MERTK also play a role in GALNT2‐mediated invasion.

## 4 Discussion

GalNAc‐type O‐glycosylation and 20 members of the GALNT gene family play crucial roles in a wide range of cellular behaviors. However, the biological and pathological roles of each GALNT member in colorectal cancers remain largely unclear. In this study, we focused on *GALNT2*, the best GALNT gene associated with poor survival of colorectal cancer patients in public databases. We showed that GALNT2 promoted colon cancer cell migration and invasion *in vitro* and peritoneal metastasis *in vivo*. By contrast, silencing of GALNT2 suppressed these malignant behaviors. Mechanistically, we demonstrated that AXL, an emerging target for cancer therapy, was a novel protein substrate of GALNT2 and GALNT2 regulated AXL degradation primarily through the proteasome‐dependent pathway. Moreover, the GALNT2‐mediated invasion of colon cancer cells was almost blocked by AXL inhibitors or siRNAs, suggesting that AXL is a crucial determinant in the GALNT2‐mediated invasiveness. The information gained from this study provides novel insights into the role of GALNT2‐AXL axis in the pathogenesis of colorectal cancer progression.

AXL expression is associated with increased risk of metastasis and poor survival in a variety of solid cancers, including breast cancer, non‐small‐cell lung adenocarcinoma, ovarian cancer, clear cell renal carcinoma, and pancreatic cancer [28, 29, 30, 31, 32]. In colorectal cancer, AXL was found to be overexpressed in liver and peritoneal metastatic tumors [33] and was a prognostic biomarker of poor overall survival in patients with early‐stage colorectal cancer [34]. AXL is a predictor of poor survival and of resistance to anti‐EGFR therapy in RAS wild‐type metastatic colorectal cancer [35]. Moreover, AXL was shown as a major regulator of migration, invasion, and epithelial–mesenchymal transition (EMT) in colon cancer cells. AXL is also expressed and plays a key role in fibroblasts, vascular cells, and several immune cells [27]. Therefore, AXL is an emerging target for cancer therapy. Consistent with previous reports suggesting a crucial role of AXL in cancer cell migration, invasion, and metastasis, our results showed that GALNT2‐mediated invasion was significantly reversed by inhibiting AXL expression with pharmacologic or genetic approach. We, therefore, proposed that GALNT2‐mediated invasiveness of colorectal cancer is at least partly through AXL. We have also found that GALNT2 was able to mildly regulate the phosphorylation of EGFR and MET in colon cancer cells. Moreover, TYRO3 and MERTK also contribute to GALNT2‐mediated invasion. Although detailed mechanisms of how GALNT2 regulates TYRO3 and MERTK remain to be further investigated, it is reasonable to expect that all TAM receptors share similar functions and could involve in GALNT2‐mediated invasive behaviors. Their relative contributions to cellular invasiveness may depend on their expression levels in colon cancer cells. Our findings support that GALNT2 modulates cancer cell behaviors by activating multiple RTKs with varying degrees. Here, we found that GALNT2 knockdown suppressed the activity of EGFR and MET in colon cancer cells. In contrast, our previous reports showed that GALNT2 knockdown enhanced their activity in gastric cancer cells. It has been reported that GALNT isoforms exhibit differential acceptor substrate specificities toward peptides and glycopeptides with GalNAc [36]. In another word, sequence compositions of and prior GalNAc moieties on a peptide determine the O‐glycosites initiated by individual GALNTs. One of the possible scenarios could be that GALNT2 initiates O‐glycosylation on specific Ser/Thr residues influenced by the prior GalNAc moieties on EGFR or MET. In different cell types, the synthesis of O‐glycans on a protein is initiated by a specific repertoire of GALNT enzymes. Thus, the O‐glycosylation sites on EGFR and MET initiated by GALNT2 could vary in different cancer types, which leads to differential effects of GALNT2 on the activities of EGFR and MET. The second explanation could be that GALNT2 initiates O‐glycosylation at the same sites on EGFR and MET, but the glycosyltransferases for further modification of the O‐glycans are differentially expressed in different cancer types. Therefore, the different O‐glycan structures on EGFR and MET cause differential effects on their activities.

We found that GALNT2‐mediated O‐glycosylation regulates AXL protein stability through the proteasomal degradation pathway. In line with our findings, glycosylation has been reported to regulate the proteasomal degradation of transmembrane receptors. Abnormal N‐glycosylation of PD‐L1 triggered by D‐mannose treatment promotes its proteasomal degradation [37]. The T168I mutation of CSF3R causes loss of O‐glycosylation at T168 [38] and the T168I CSF3R mutant undergoes enhanced degradation via the proteasome [39]. Interestingly, the proteasome‐dependent degradation of full‐length AXL (AXL‐FL) has been reported to couple with production of AXL intracellular domain (ICD), and AXL‐ICD could regulate the expression of cancer‐related genes [40], which is believed to be one of the ligand‐independent functions of AXL. With respect to whether GALNT2 can regulate ligand‐dependent activation of AXL, unfortunately, we could not detect sufficient amount of phospho‐AXL in colon cancer cells to draw a conclusion. This question remains to be answered in other cancer types where high phospho‐AXL levels are present. To extend our findings, it will be of great interest to identify the precise O‐glycosylation sites on AXL and investigate how the O‐glycans regulate AXL properties such as phosphorylation, subcellular localization, and heterodimerization with other RTKs. We found that inhibition of O‐glycosylation with benzyl‐α‐GalNAc resulted in lower molecular weight of AXL. However, GALNT2 depletion reduced the total level of AXL without notable changes in the molecular weight of AXL. The reason could be that there are multiple O‐glycosylation sites on AXL, but GALNT2 only initiates O‐glycosylation on a part of these sites. Therefore, it is highly possible that other GALNT enzymes can also glycosylate AXL in colon cancer cells. Moreover, the TAM members AXL, TYRO3, and MER share similar structures. Indeed, we found that all TAM receptors carry O‐glycans. It is reasonable to expect that O‐glycosylation plays a critical role in regulating the functions of all TAM receptors. Our findings open novel insights into the significance of GalNAc‐type O‐glycosylation in TAM receptor biology, which is important for cancer, cardiovascular, autoimmune, and neuronal diseases [4].

## 5 Conclusions

In this study, GALNT2 is overexpressed in colorectal tumors and correlates with poor prognosis. We demonstrated that TAM receptors are O‐glycosylated and GALNT2 promotes invasive behaviors of colon cancer cells partly through modification of O‐glycosylation and protein stability of AXL. This study highlights the functional significance of GalNAc‐type O‐glycosylation in TAM receptors.

## Conflict of interest

The authors declare no conflict of interest.

## Author contributions

JH and J‐SH collected clinical samples and analyzed data. Y‐YL, Y‐TC, T‐WH, H‐YL, S‐CH and S‐TC conducted the experiments and analyzed the data. M‐CH and Y‐YL wrote the manuscript. N‐YL, H‐JY, J‐TL, JH and M‐CH contributed to the project's conception. All authors approved the final version of the article including the authorship list.

### Peer review

The peer review history for this article is available at https://publons.com/publon/10.1002/1878‐0261.13347.

## Supporting information


**Table S1.** Demographic data and clinicopathological details of the study patients (*n* = 58).

**Fig. S1.** Effects of GALNT2 on cell viability using MTT assays.
**Fig. S2.** GALNT2 knockdown suppresses migration and invasion of SW480 cells.
**Fig. S3.** GALNT2 knockout weakly decreases phosphorylation of EGFR and MET.
**Fig. S4.** Inhibition of EGFR or MET activity cannot block GALNT2‐mediated migration and invasion of HCT116 cells.
**Fig. S5.** TYRO3 and MERTK carry GalNAc‐type O‐glycans.
**Fig. S6.** Real‐time RT‐PCR analysis showing effects of GALNT2 on AXL mRNA levels.
**Fig. S7.** Effects of GALNT2 on GAS6‐mediated phosphorylation of AXL.
**Fig. S8.** AXL inhibitor TP‐0903 suppresses and reverses GALNT2‐mediated invasion of colon cancer cells.
**Fig. S9.** Effects of TYRO3 or MERTK knockdown on GALNT2‐mediated invasion of HCT116 cells.
**Fig. S10.** GALNT2 knockdown or knockout decreases TYRO3 and MERTK protein levels.

## Data Availability

The data that support the findings of this study are available in the Supporting Information of this article (Figs S1–S10 and Table S1).
