# Supplementary material for: GALNT2 promotes invasiveness of colorectal cancer cells partly through AXL
Source: Mol Oncol. 2022 Dec 5;17(1):119–33. doi: 10.1002/1878-0261.13347 (PMC9812829; doi:10.1002/1878-0261.13347)
Supplement: Supplementary file 1 — Table S1. Demographic data and clinicopathological details of the study patients (n = 58). [file MOL2-17-119-s002.docx]

**Supplementary Table S1. Demographic data and clinicopathological details of the study patients (*n* = 58).**

| **Variables** | **Patient number n* (%)** |
| --- | --- |
| **Age**, mean ± SD, years | 66.16 ± 13.92 |
| **Gender** |  |
| Male | 28 (48.3) |
| Female | 30 (51.7) |
| **Clinical stage** |  |
| I | 5 (9.1) |
| II | 9 (16.4) |
| III | 39 (70.9) |
| IV | 2 (3.6) |
| **T status** |  |
| T_1-2_ | 22 (40.0) |
| T_3_ | 17 (30.9) |
| T_4_ | 16 (29.1) |
| **Nodal metastasis** |  |
| N_0_ | 27 (48.2) |
| N_1-2_ | 29 (51.8) |
| **Distant metastasis** |  |
| M_0_ | 16 (28.6) |
| M_1_ | 40 (71.4) |
| **Histologic grade** |  |
| Low grade | 46 (93.9) |
| High grade | 3 (6.1) |
| **Overall free survival months** |  |
| Mean ± SD | 11.5 ± 9.37 |
| Max | 32 |
| Min | 1 |

*Some patients do not have information on certain variables.
