# Supplementary material for: GALNT2 promotes invasiveness of colorectal cancer cells partly through AXL
Source: Mol Oncol. 2022 Dec 5;17(1):119–33. doi: 10.1002/1878-0261.13347 (PMC9812829; doi:10.1002/1878-0261.13347)
Supplement: Supplementary file 2 — Fig. S1. Effects of GALNT2 on cell viability using MTT assays. Fig. S2. GALNT2 knockdown suppresses migration and invasion of SW480 cells. Fig. S3. GALNT2 knockout weakly decreases phosphorylation of EGFR and MET. Fig. S4. Inhibition of EGFR or MET activity cannot block GALNT2‐mediated migration and invasion of HCT116 cells. Fig. S5. TYRO3 and MERTK carry GalNAc‐type O‐glycans. Fig. S6. Real‐time RT‐PCR analysis showing effects of GALNT2 on AXL mRNA levels. Fig. S7. Effects of GALNT2 on GAS6‐mediated phosphorylation of AXL. Fig. S8. AXL inhibitor TP‐0903 suppresses and reverses GALNT2‐mediated invasion of colon cancer cells. Fig. S9. Effects of TYRO3 or MERTK knockdown on GALNT2‐mediated invasion of HCT116 cells. Fig. S10. GALNT2 knockdown or knockout decreases TYRO3 and MERTK protein levels. [file MOL2-17-119-s001.pdf]

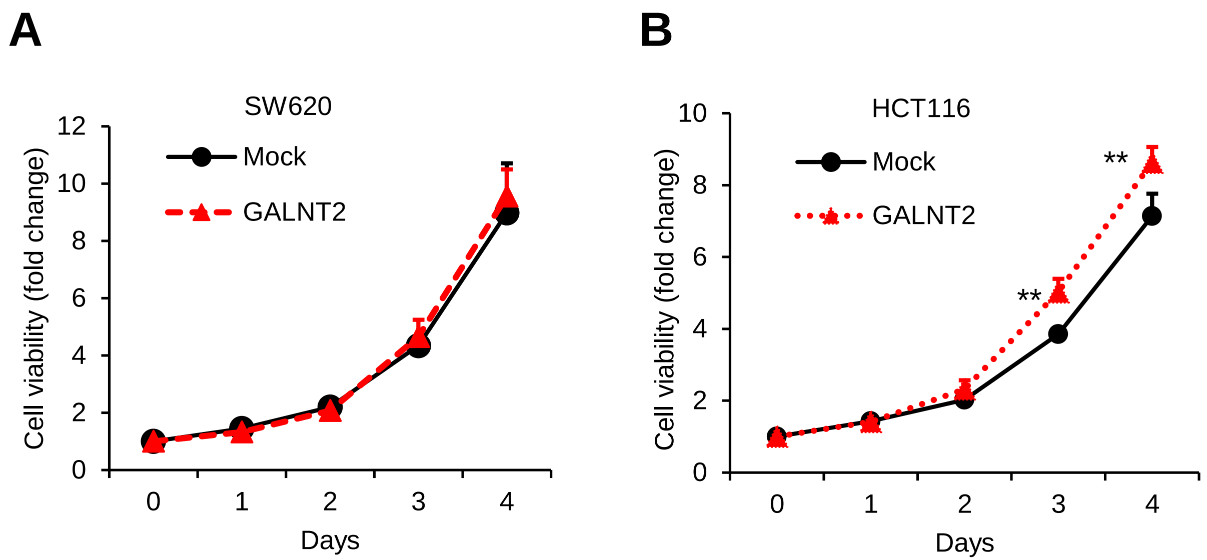

Supplementary Figure S1. Effects of GALNT2 on cell viability using MTT assays. A. Effects of GALNT2 overexpression on viability of SW620 cells. **B.** Effects of GALNT2 overexpression on viability of HCT116 cells. $^{**}P < 0.01$.

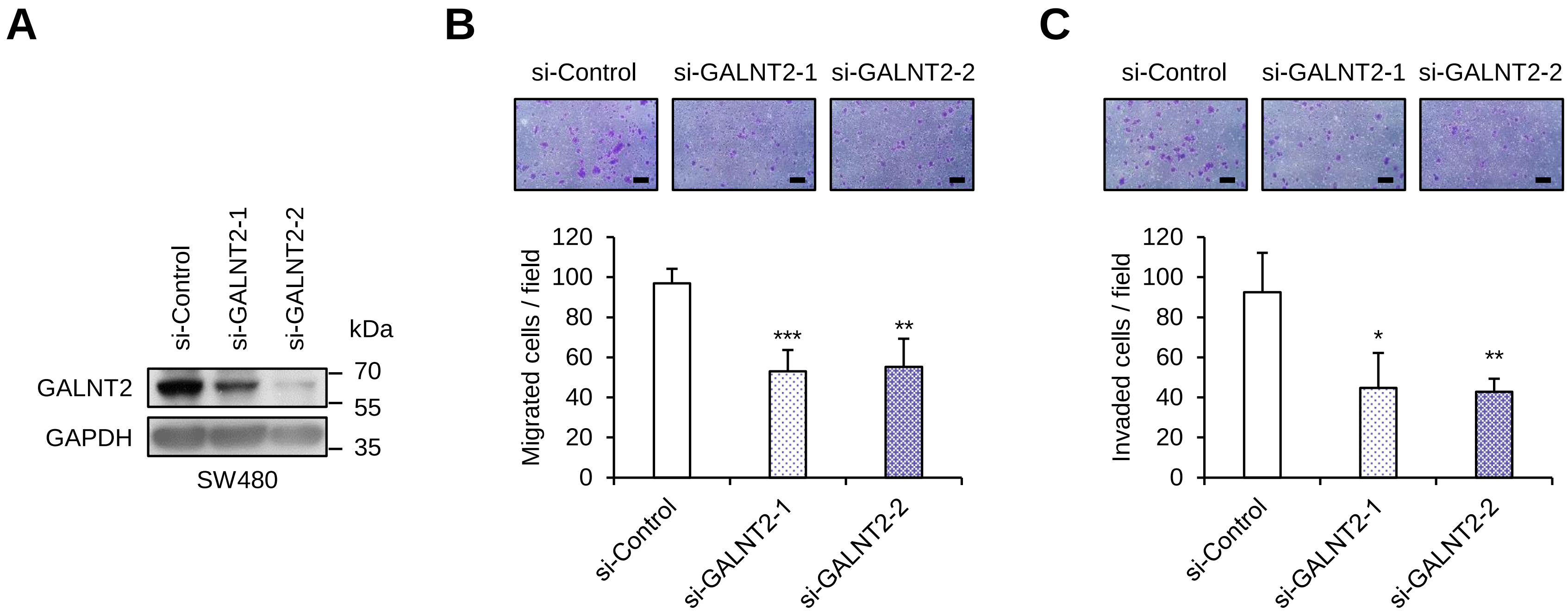

Supplementary Figure S2. GALNT2 knockdown suppresses migration and invasion of SW480 cells. A. Western blots showing knockdown of GALNT2 with siRNAs in SW480 cells. **B.** Cell migration was analyzed using transwell migration assay. **C.** Cell invasion was analyzed using Matrigel invasion assay. Cell migration and invasion were triggered by 10% FBS for 48h. Representative images were shown. Scale bar, 20 µm. Data are presented as mean ± SD. *$P < 0.05$, **$P < 0.01$, ***$P < 0.001$.

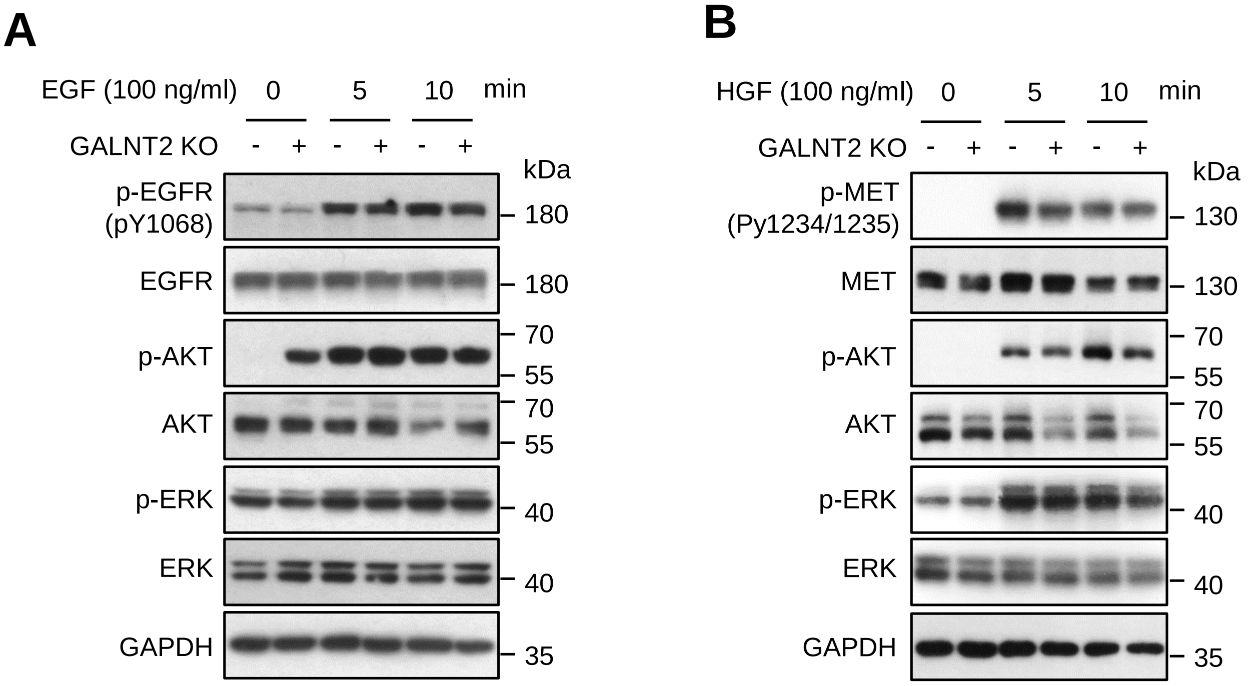

Supplementary Figure S3. GALNT2 knockout weakly decreases phosphorylation of EGFR and MET. A. Western blots showing effects of GALNT2 knockout on EGFR signaling. Parental and GALNT2 knockout (KO) HCT116 cells were starved overnight and then treated with 100 ng/ml EGF. **B.** Western blots showing effects of GALNT2 knockout on MET signaling. Parental and GALNT2 knockout (KO) HCT116 cells were starved overnight and then treated with 100 ng/ml HGF.

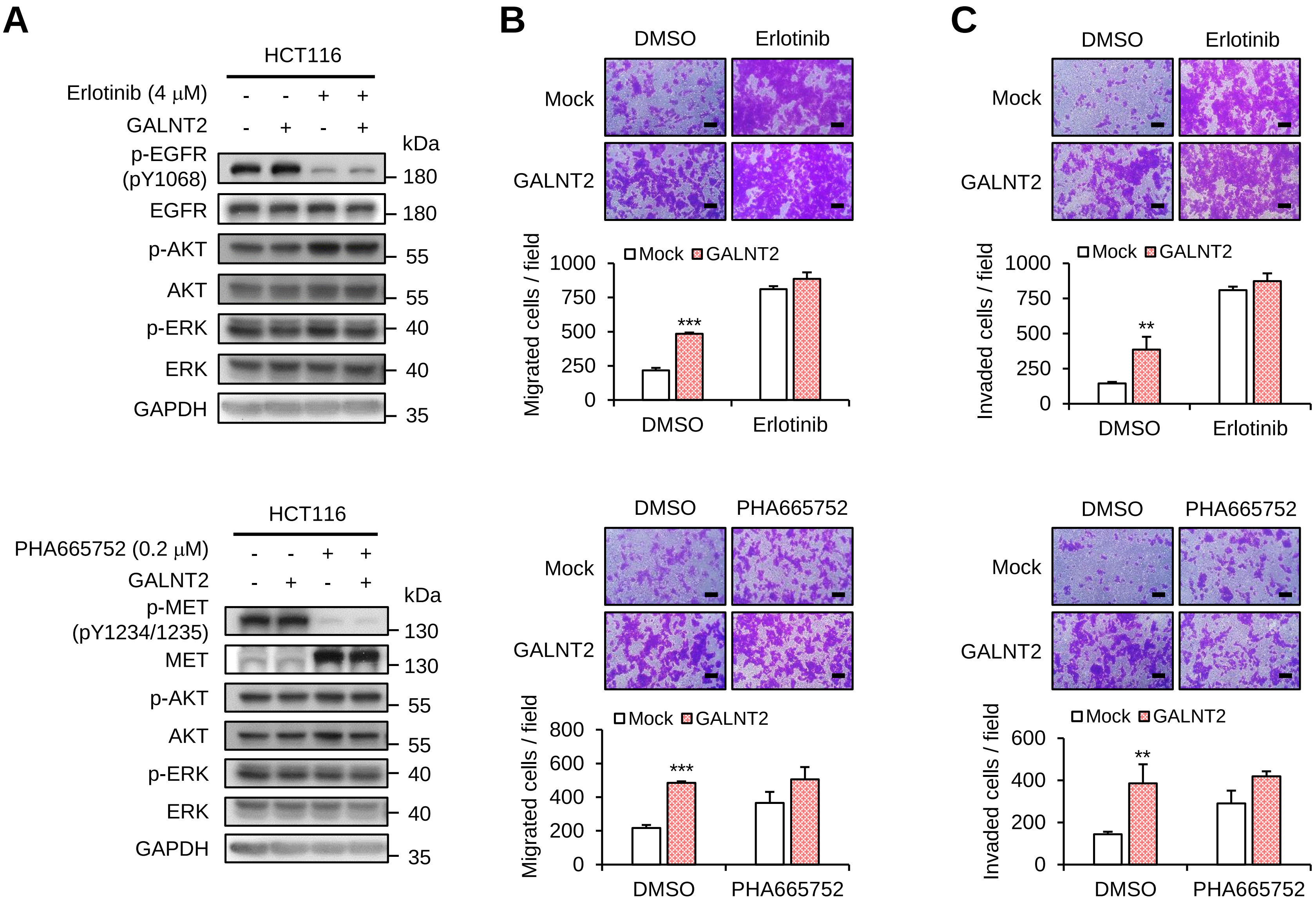

Supplementary Figure S4. Inhibition of EGFR or MET activity cannot block GALNT2-mediated migration and invasion of HCT116 cells. A. Western blots showing effects of erlotinib and PHA665752 on EGFR and MET phosphorylation and signaling, respectively. **B.** Transwell migration assay. **C.** Matrigel invasion assay. Cell migration and invasion were triggered by 10% FBS for 48h. Representative images were shown. Scale bar, 20 μm. Data are presented as mean ± SD. $^{**}P < 0.01$, $^{***}P < 0.001$.

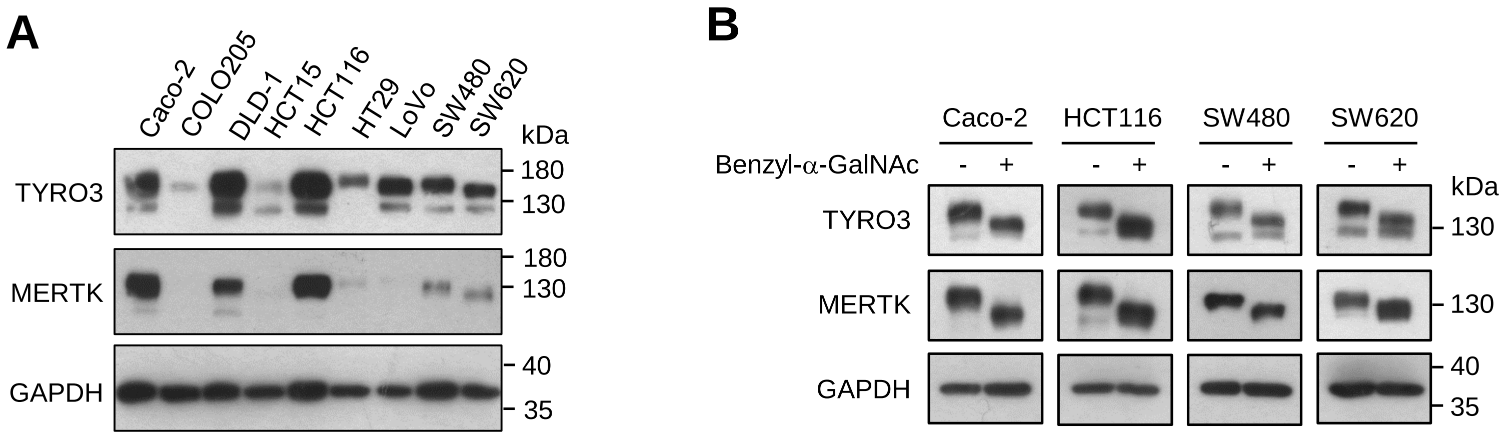

Supplementary Figure S5. TYRO3 and MERTK carry GalNAc-type O-glycans. A. Western blots showing expression of TYRO3 and MERTK in colon cancer cells. **B.** Western blots showing a dramatic decrease in the molecular weight of TYRO3 and MERTK in cells treated with O-glycan synthesis inhibitor 1 mM benzyl-α-GalNAc for 24 h.

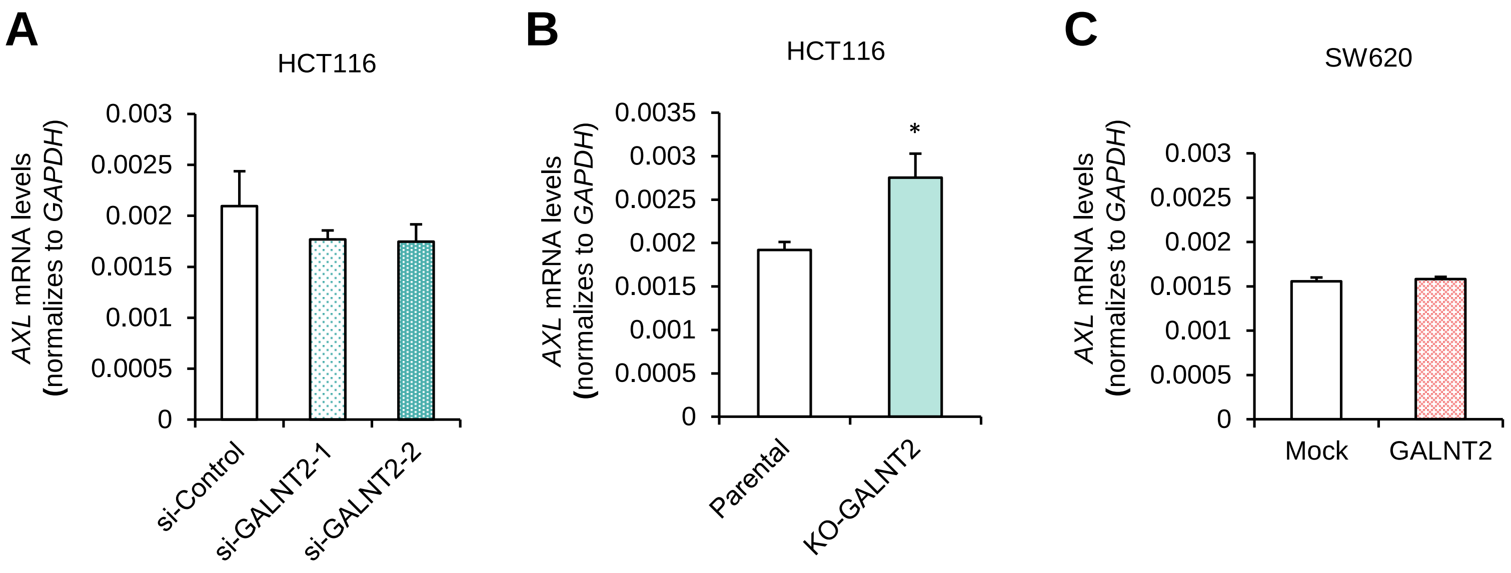

Supplementary Figure S6. Real-time RT-PCR analysis showing effects of GALNT2 on *AXL* mRNA levels. A. Effects of GALNT2 knockdown on *AXL* mRNA expression in HCT116 cells. **B.** Effects of GALNT2 knockout (KO) on *AXL* mRNA expression in HCT116 cells. **C.** Effects of GALNT2 knockdown on *AXL* mRNA expression in SW620 cells. *$P < 0.05$.

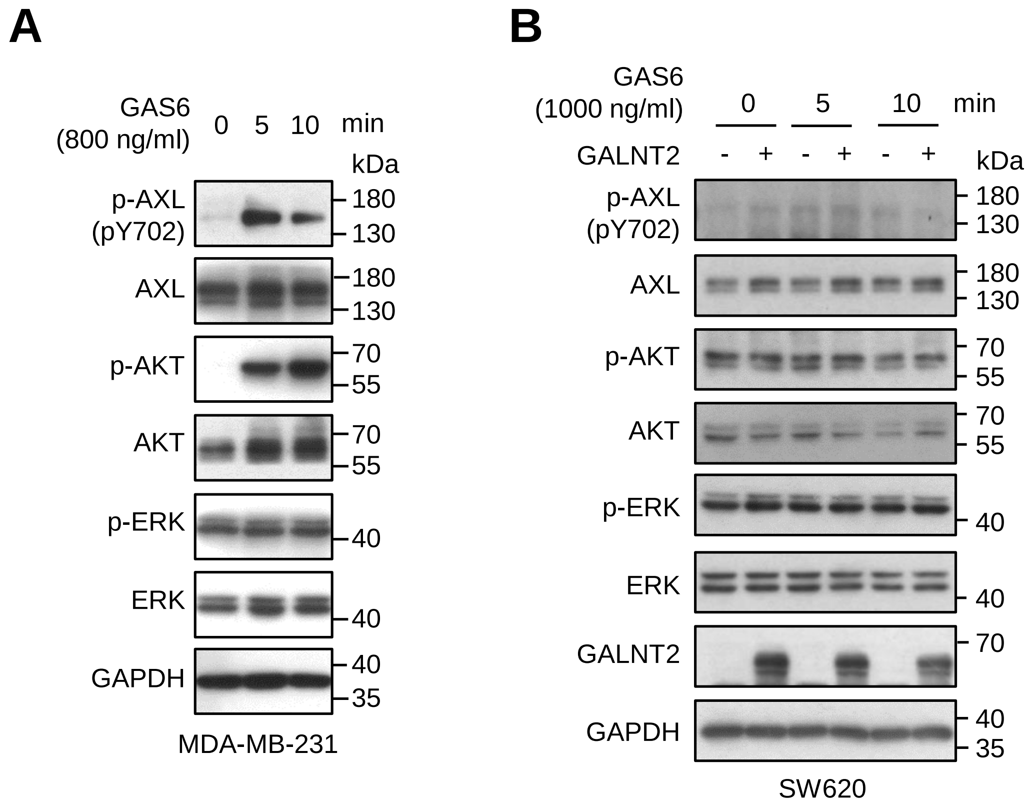

Supplementary Figure S7. Effects of GALNT2 on GAS6-mediated phosphorylation of AXL. A. Western blots showing GAS6-mediated phosphorylation of AXL in breast cancer cell line MDA-MB-231. All cells were starved overnight and then treated with GAS6 for different times, as indicated. **B.** Western blots showing effects of GALNT2 overexpression on GAS6-mediated phosphorylation of AXL in SW620 cells.

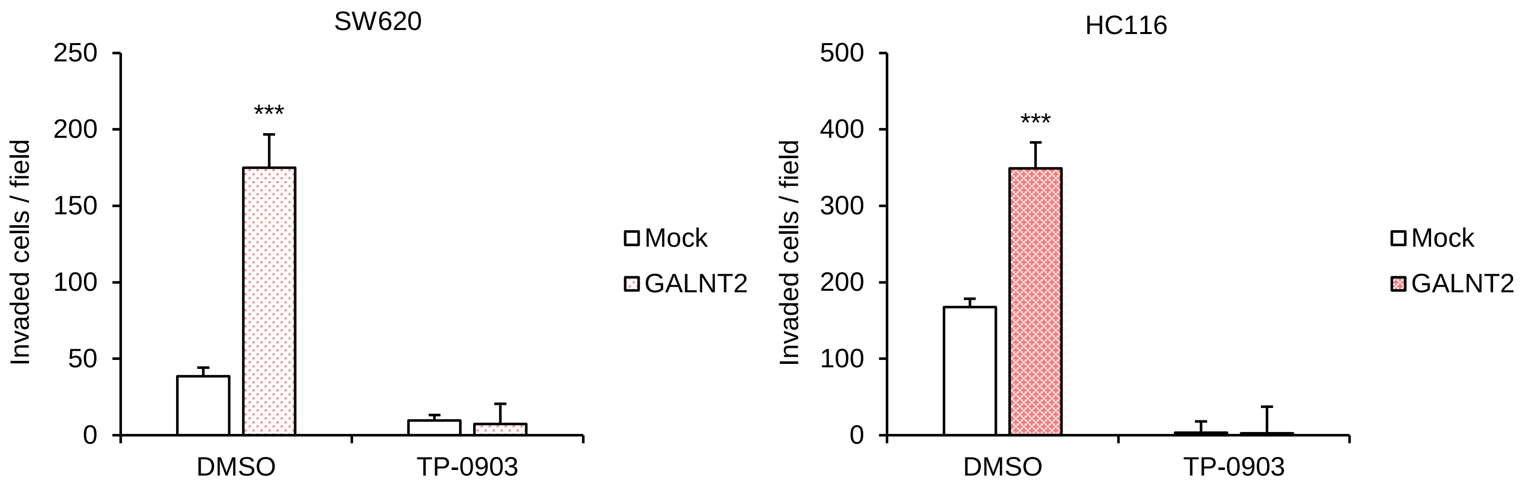

Supplementary Figure S8. AXL inhibitor TP-0903 suppresses and reverses GALNT2- mediated invasion of colon cancer cells . Mock or GALNT2 overexpressing SW620 and HCT116 cells were used. Cell invasion was triggered by 10% FBS together with DMSO solvent control or 3 μM TP-0903, an AXL inhibitor, in the lower chamber for 48 h. ***P < 0.001.

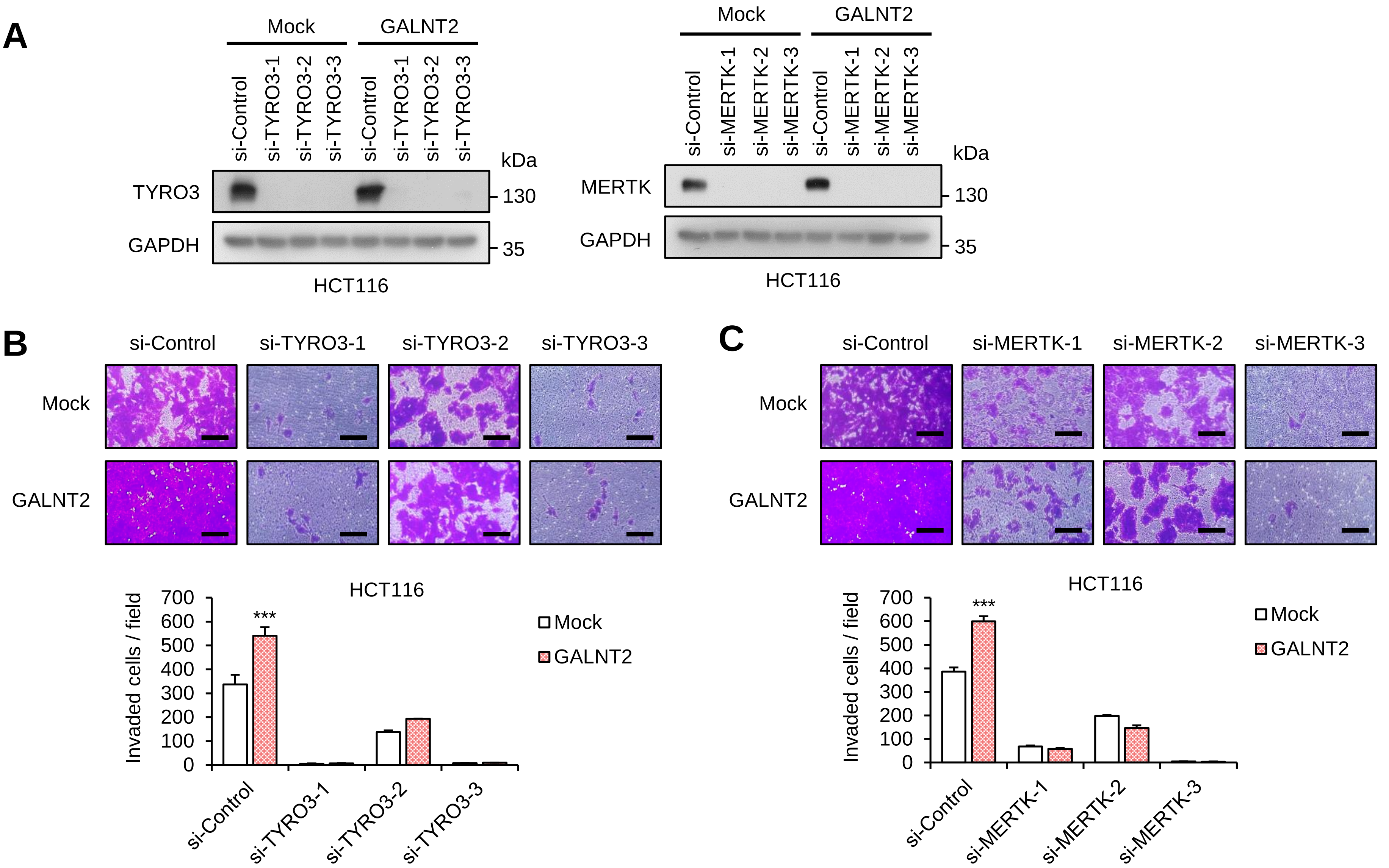

Supplementary Figure S9. Effects of TYRO3 or MERTK knockdown on GALNT2-mediated invasion of HCT116 cells. A. Western blots showing siRNA-mediated knockdown of TYRO3 or MERTK in HCT116 cells. **B.** Effects of TYRO3 knockdown on HCT116 cell invasion analyzed using Matrigel invasion assay. Representative images were shown. ****P* < 0.001. **C.** Effects of MERTK knockdown on HCT116 cell invasion analyzed using Matrigel invasion assay. Cell invasion was triggered by 10% FBS for 48h. Representative images were shown. Scale bar, 20 μm. Data are presented as mean ± SD. ****P* < 0.001.

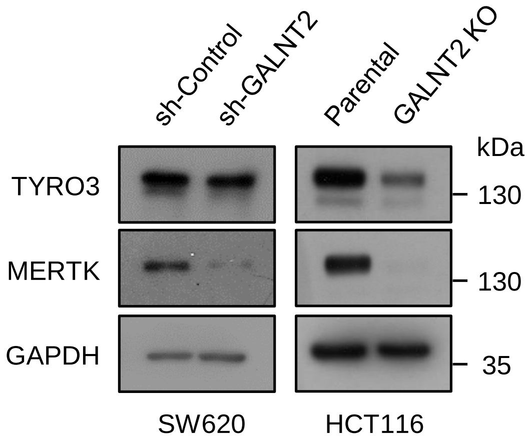

Supplementary Figure S10. GALNT2 knockdown or knockout decreases TYRO3 and MERTK protein levels. Western blots showing expression of TYRO3 and MERTK in control or GALNT2 knockdown SW620 cells as well as parental or GALNT2 knockout (KO) HCT116 cells. GAPDH was an internal control.
